# Comparative Genomics of *Pseudomonas stutzeri* Complex: Taxonomic Assignments and Genetic Diversity

**DOI:** 10.3389/fmicb.2021.755874

**Published:** 2022-01-13

**Authors:** Xiangyang Li, Zilin Yang, Zhao Wang, Weipeng Li, Guohui Zhang, Hongguang Yan

**Affiliations:** ^1^School of Sciences, Kaili University, Kaili, China; ^2^Bacterial Genome Data Mining and Bioinformatic Analysis Center, Kaili University, Kaili, China; ^3^School of Life and Health Science, Kaili University, Kaili, China; ^4^School of Big Data Engineering, Kaili University, Kaili, China

**Keywords:** genome comparison, taxonomy and nomenclature, phylogeny, genetic diversity, pan-genome, *Psuedomonas stutzeri* complex

## Abstract

*Pseudomonas stutzeri* is a species complex with extremely broad phenotypic and genotypic diversity. However, very little is known about its diversity, taxonomy and phylogeny at the genomic scale. To address these issues, we systematically and comprehensively defined the taxonomy and nomenclature for this species complex and explored its genetic diversity using hundreds of sequenced genomes. By combining average nucleotide identity (ANI) evaluation and phylogenetic inference approaches, we identified 123 *P. stutzeri* complex genomes covering at least six well-defined species among all sequenced *Pseudomonas* genomes; of these, 25 genomes represented novel members of this species complex. ANI values of ≥∼95% and digital DNA-DNA hybridization (dDDH) values of ≥∼60% in combination with phylogenomic analysis consistently and robustly supported the division of these strains into 27 genomovars (most likely species to some extent), comprising 16 known and 11 unknown genomovars. We revealed that 12 strains had mistaken taxonomic assignments, while 16 strains without species names can be assigned to the species level within the species complex. We observed an open pan-genome of the *P. stutzeri* complex comprising 13,261 gene families, among which approximately 45% gene families do not match any sequence present in the COG database, and a large proportion of accessory genes. The genome contents experienced extensive genetic gain and loss events, which may be one of the major mechanisms driving diversification within this species complex. Surprisingly, we found that the ectoine biosynthesis gene cluster (*ect*) was present in all genomes of *P. stutzeri* species complex strains but distributed at very low frequency (43 out of 9548) in other *Pseudomonas* genomes, suggesting a possible origin of the ancestors of *P. stutzeri* species complex in high-osmolarity environments. Collectively, our study highlights the potential of using whole-genome sequences to re-evaluate the current definition of the *P. stutzeri* complex, shedding new light on its genomic diversity and evolutionary history.

## Introduction

*Pseudomonas stutzeri* is a species complex comprising five defined species, *P. stutzeri* (sensu stricto) (s.s.), *P. xanthomarina* (Romanenko et al., 2005), *P. balearica* (Bennasar et al., 1996), *P. luteola* and *P. chloritidismutans* (Wolterink et al., 2002), according to the National Center for Biotechnology Information (NCBI) Taxonomy Database. Strains belonging to *P. stutzeri* complex display extremely broad phenotypic diversity and consequently colonize a large range of ecological niches (Lalucat et al., 2006). Although rarely causing infections, over the past two decades, *P. stutzeri* complex members have been increasingly recognized as a cause of infection in humans (Scotta et al., 2012; Hequette-Ruz et al., 2018; Halabi et al., 2019). The *P. stutzeri* complex has received particular attention because of specific physiological properties, such as denitrification (Pena et al., 2012), nitrogen fixation (Lalucat et al., 2006; Yan et al., 2008; Busquets et al., 2013; Paerl et al., 2018), degradation of aromatic compounds (Brunet-Galmes et al., 2012; Busquets et al., 2013; Gomila et al., 2015a; Hirose et al., 2015; Brown et al., 2017), and metal resistance and potential application in bioremediation (Li et al., 2012; Chakraborty et al., 2017; Zheng et al., 2018; Harada et al., 2019). It was shown that one third of *P. stutzeri* complex members are naturally transformable (Sikorski et al., 2002b; Busquets et al., 2012; Smith et al., 2014), which may be one of the major driving forces causing its high level of intraspecific heterogeneity. Diversity within the species is not limited to physiological traits but is also clearly reflected at the genetic level. To date, the *P. stutzeri* complex has been divided into at least 21 defined genomovars (Scotta et al., 2013), a provisional taxonomic status for genotypically similar strains within a bacterial species. However, there is lack of defined phenotypic properties for discriminating among different genomovars (Lalucat et al., 2006). Despite focus on its genetic heterogeneity and taxonomy, most investigations of *P. stutzeri* have been performed on the basis of DNA-DNA hybridization (DDH), 16S rRNA gene similarity, and sequencing of several housekeeping genes (multilocus sequence analysis, MLSA) (Guasp et al., 2000; Scotta et al., 2013; Zhang et al., 2014). With advances in high-throughput sequencing technology, numerous genomes representing this group of strains have been determined. However, a comprehensive, genome-wide analysis of this species complex related to its diversity and taxonomy is lacking.

The DDH, 16S rRNA gene analysis and MLSA are the most frequently used approaches for assigning isolates to species within the *P. stutzeri* complex. All genomovars of the species complex described so far have been described using these approaches (Rossello et al., 1991; Sikorski et al., 2005; Gomila et al., 2015b). However, each of these techniques has some basic limitations. DDH is the gold standard for prokaryotic species circumscriptions, and construction of the existing prokaryotic classification system was based on the fact that DDH can reveal coherent genomic groups (genospecies) of strains generally sharing DDH values with > 70% similarity (Colston et al., 2014). However, laboratory-based DDH measurements are complex and time-consuming. Therefore, the difficulty of obtaining DDH values for a large number of genomes is a major drawback in the bioinformatics era. Similarly, the low variability and conservative nature of 16S rRNA genes gives rise to insufficient resolution for inferring clear taxonomic relationships (Rossi-Tamisier et al., 2015), while putative bias in the selection of genes is a major drawback for MLSA.

Genome sequencing has become a routine practice in microbiology research due to advances in high-throughput sequencing technology and declining costs of sequencing. At the time of writing, there are more than 250,000 prokaryotic genomes that have been released in the NCBI genome database. As a result, new tools are being developed to assess taxonomic relationships and discover novel taxa, primarily digital DNA-DNA hybridization (dDDH) (Chain et al., 2009; Meier-Kolthoff et al., 2013) and average nucleotide identity (ANI) (Richter and Rossello-Mora, 2009). Taxonomists already believe that the reference standard for determining taxonomy will be full genome sequences. Digital DNA-DNA hybridization produces values that compare closely with experimentally derived DDH values (Auch et al., 2010; Meier-Kolthoff et al., 2013). Average nucleotide identity between two genomes has been proposed as a promising standard for defining prokaryotic species and is receiving wide acceptance (Federhen et al., 2016; Ciufo et al., 2018). The most recent proposal recommends using an ANI threshold of 94∼96% (Konstantinidis and Tiedje, 2005; Jain et al., 2018), along with support from tetranucleotide frequency correlation coefficient (TETRA) values > 0.99 (Richter and Rossello-Mora, 2009).

An increasing number of *P. stutzeri* complex genomes have been obtained from a variety of ecosystems (listed in Supplementary Table 1), providing researchers with an opportunity to extensively analyze genetic diversity, assess taxonomic relationships, discover novel taxa and identify genetic determinants associated with specific physiological properties. In this study, by coupling a large complete genome collection with powerful bioinformatics analyses, we documented taxonomic and phylogenetic relationships in this species complex and elaborated on its diversity at the genomic level. Finally, we surveyed the distribution of the ectoine biosynthesis gene cluster (*ect*) in all *Pseudomonas* genomes to inquire into a possible origin of the ancestors of the *P. stutzeri* complex in high-osmolarity habitats. Our results underlined the misidentifications for *P. stutzeri* complex in taxonomy and nomenclature, and revealed that a substantial proportion of pan-genome has undergone gene loss and gain, essentially shaping the genetic diversity of the *P. stutzeri* complex.

## Materials and Methods

### Genomic Sequences of *Pseudomonas stutzeri* Species Complex

*Pseudomonas stutzeri* has a complex taxonomy encompassing several species. To define *P. stutzeri* complex genomes, a combination of ANI and MLSA approaches were used to sieve *P. stutzeri* complex genomes from all sequenced *Pseudomonas* genomes. All available 9,548 *Pseudomonas* genomes (as of 6 February 2020) were retrieved from the NCBI genome database^1^ using Aspera.^2^ First, 9,548 *Pseudomonas* genomes were queried against 105 genomes of the *P. stutzeri* group (Supplementary Table 2) in NCBI for ANI calculation using fastANI version 1.3 (Jain et al., 2018). Then, genomes having ≥ 80% identity by ANI to reference genomes were retained for curated ANIm calculation using Jspecies version 1.2.1 (Richter and Rossello-Mora, 2009) with default parameters. ANI values were transformed into a “100%-ANI” distance matrix and clustered using MEGAX version 10.0.4 (Kumar et al., 2018) with the neighbor-joining (NJ) algorithm. Simultaneously, three housekeeping genes (16S rRNA, *gyrB* and *rpoD* partial gene sequences) were extracted from the *Pseudomonas* genomes having ≥ 80% identity by ANI to reference genomes. Besides, these three genes of 53 well-characterized *P. stutzeri* complex strains were downloaded from NCBI nucleotide database according to a previous study (Scotta et al., 2013). A NJ phylogenetic tree based on the concatenated analysis of the three genes was generated utilizing MEGAX. Finally, only genomes located in the same branch with the well-characterized (or defined) *P. stutzeri* complex strains in both ANI and MLSA analyses were considered to be members of the *P. stutzeri* complex.

### Digital DNA-DNA Hybridization

Estimation of dDDH was performed using the Genome-to-Genome Distance Calculator (GGDC) (Chain et al., 2009; Meier-Kolthoff et al., 2013). Accession numbers of *P. stutzeri* complex genomes were submitted to the GGDC 2.0 Web server,^3^ where dDDH calculations were performed. Results of GGDC were used for analysis according to formula 2, which calculates dDDH estimates independent of genome length and is recommended by the authors of GGDC for use with draft genomes (Chain et al., 2009; Meier-Kolthoff et al., 2013).

### Determination of Core- and Pan-Genomes

All predicted protein-coding sequences (CDSs) were extracted from each of 123 genomes separately using extract_protein_dir.pl^4^ and subjected to identification of homologous clusters using OrthoFinder version 2.3.12 (Emms and Kelly, 2019) with default parameters. This tool is fast, accurate and scalable to numerous genomes compared with OrthoMCL. Results for orthogroups and unassigned genes (strain-specific genes) from OrthoFinder were combined, and the combined data format was modified using format_modification.pl.^5^ These data were subsequently used to estimate the core- and pan-genomes for a given number of sequentially added genomes *via* the PGAP tool v1.2.1 (Zhao et al., 2012). Sample size was set to 20,000 according to recommendations for this software. The core genome was defined as homologous genes shared by all genomes analyzed. The pan-genome was defined as the non-redundant set of all genes found in all genomes analyzed.

In addition, the percentage of homologs shared between pairs of genomes was used to reflect the genetic diversity in their gene content. This was defined as the number of shared homologs divided by the average number of genes in the non-redundant set of all CDSs between two genomes. The number of shared homologs for any pair of genomes and the number of genes in the non-redundant set of all CDSs for each genome were obtained from the results files “Comparative Genomics Statistics Directory” generated by Orthofinder. In total, 15,129 pairwise values were calculated for 123 genomes.

### Phylogenetic Tree Inference

Core genes were obtained using OrthoFinder. Single-copy core genes, which are shared by all genomes and present as only a single copy in each genome, were used to infer phylogenetic relationships among 123 *P. stutzeri* complex strains plus four outgroup strains (*Pseudomonas aeruginosa* DSM 50071^T^, *P. azotifigens* DSM 17556^T^, *P. mendocina* NCTC10897^T^ and *P. putida* FF4). All nucleotide alignments of core genes were generated using Muscle version 3.8.31 (Edgar, 2004), and core gene sequences were concatenated into a string of nucleotides for each genome using Concat_Seq.pl.^6^ The concatenated alignment data were used to infer phylogenies using MEGAX version 10.0.4 with the NJ algorithm, and RAxML version 8.2.11 (Stamatakis, 2014) with the maximum-likelihood (ML) algorithm under the general time-reversible (GTR) + category (CAT) model. To estimate tree reliability, a bootstrap method computing 1,000 bootstrap repetitions was used for both the NJ and ML trees. In addition, GGDC distance (intergenomic distance) matrices were used to construct a tree using FastME version 2.1.5 (Lefort et al., 2015). Statistical comparisons of tree topologies were made using the Kendall-Colijn metric implemented through the R package treespace (Jombart et al., 2017). For all pairwise tree comparisons, trees were rooted to four outgroup strains, and a lambda value of 0 (to give weight to tree topology rather than branch lengths) was used along with 100,000 random trees as a background distribution. A z-test was performed comparing the distance observed between the query and the reference to the distances observed between the query and the trees from the background distribution. A threshold of *P* < 0.05 was used to determine that the query tree is more closely related to the reference tree topology than would be expected by chance. All trees were visualized using ITOL (Letunic and Bork, 2019).

### Dynamics of Gene Content Analysis

Gain and loss of gene families at each ancestral node were modeled using Count v10.04 (Csuros, 2010) using the Pan-genome matrix of 123 genomes and a modified core-gene based ML tree as input files. This tree was generated by removing the outgroup strains of the core-gene based ML tree using iTOL. All of the model parameters were first optimized by maximizing the likelihood of the data using a gain–loss-duplication model with a Poisson distribution for gene family size at the root. Gamma-distributed rate variation across gene families was assumed, with the shape parameter discretized in four classes; a fixed gain/loss ratio across lineages was also assumed. Rate parameters were optimized after 100 rounds of parameter optimization. Profiles of posterior probabilities of events for each branch of the tree were then estimated. To obtain patterns of gain/loss, these probabilities were transformed into “likely events” using a threshold of 0.5 posterior probability (Brito et al., 2018).

### Functional Analysis

We extracted the amino acid sequences of the unique genes and the first genes for each set of homologs in the pan-genome. To characterize the functional classification of pan-genome, these sequences were queried against the Clusters of Orthologous Groups of proteins (COGs, v2021) database (Galperin et al., 2021) using blastP (Boratyn et al., 2013) with E-value of 1e-5 (Zhong et al., 2021). COG_annotation.pl^7^ was used to perform the blast analysis and classified the function of the genes according to COG classifications. If a gene was assigned to more than one COG category, each COG category was calculated separately.

### Origin of the Cloud Genome

The cloud genome was defined as homologous protein clusters shared by only one or two genomes. To identify potential donor species of the cloud genome, all cloud genes were queried against the NCBI nr database using a blastp search with an E-value of 1e-5. The blastp hits excluded sequences belonging to the 123 strains analyzed in this study.

### Detection of Ectoine Biosynthesis Gene Cluster in *Pseudomonas* spp.

Ectoine biosynthesis is the common mechanism for balancing osmotic pressure for some bacteria. Biosynthesis of ectoine is catalyzed by *ectABC*, which encode N-γ-acetyltransferase, l-2,4-diaminobutyric acid transaminase and ectoine synthase, respectively. Some strains hydroxylate ectoine to 5-hydroxyectoine in a stereospecific fashion using the ectoine hydroxylase EctD. *ask_ect* is a gene encoding aspartokinase that is involved in biosynthesis of the precursor for ectoine biosynthesis, aspartyl-beta-phosphate, and enhances the biosynthesis of ectoine and hydroxyectoine (Stoveken et al., 2011). Several *P. stutzeri* complex genomes were reported to harbor complete ectoine biosynthesis gene cluster, comprising five genes (*ectABCD-ask*) (Stoveken et al., 2011; Li et al., 2012). Numerous *P. stutzeri* complex strains isolated from marine environments (Supplementary Table 1) inspired us to survey whether harboring *ectABCD-ask* is a common phenomenon in genomes of this species complex strains for surviving in salty environments (Sikorski et al., 2002a; Pena et al., 2012; Paerl et al., 2018; Zheng et al., 2018). Interested_gene_generation.pl in the Gcluster tool (Li et al., 2020) was employed to detect the distribution of the *ect* cluster among 9,548 *Pseudomonas* genomes as an initial screen. This script performs a local blastp analysis in which all protein sequences of *Pseudomonas* spp. are queried against the amino acid sequence of the ectoine synthase EctC of *P. stutzeri* A1501. In blastp analysis, subjects meeting the custom cut-off (E-value ≤ 1e-5; Identity ≥ 50%; Coverage ≥ 70%) were accepted as positive hits. All candidate genomes harboring *ectC* were visualized using Gcluster.pl (Li et al., 2020) one time and checked manually for the presence of the *ectABCD*-*ask* gene cluster.

## Results and Discussion

### Definition of *Pseudomonas stutzeri* Complex Genomes

It is well accepted that some genomes are misidentified or could be named more specifically in the NCBI database (Federhen et al., 2016; Ciufo et al., 2018). For this reason, we used two prevalent approaches, ANI analysis and phylogenetic tree reconstruction based on housekeeping genes, to identify *P. stutzeri* complex genomes among 9,548 *Pseudomonas* genomes. ANI analysis indicated that 368 of the 9,548 *Pseudomonas* genomes shared more than 80% identity by ANI with 105 genomes of *P. stutzeri* complex strains in GenBank. Cluster analysis using the “100%-ANI” distance matrix placed 133 strains in the *P. stutzeri* complex branch (Supplementary Figure 1). However, *P. stutzeri* NP_8HO was excluded in the *P. stutzeri* complex branch. The phylogenetic tree based on the concatenated analysis of the three genes (16S rRNA, *ropD* and *gyrB*) is partially incongruent with that of the cluster analysis. The disaccord is that ten strains (six *P. luteola* strains, *P. lutea* LMG 21974, *P. zeshuii* KACC 15471, and *Pseudomonas* sp. strains HPB0071 and LTJR-52) were not affiliated with the *P. stutzeri* complex branch (Supplementary Figure 2). Meanwhile, *P. luteola* strains had an average GC% of 55.02%, significantly lower than that of *P. stutzeri* complex (*P* < *0.01*, see below), supporting that *P. luteola* did not belong to the *P. stutzeri* complex. Thus, above results demonstrated that 123 genomes should be assigned to the *P. stutzeri* complex, and they are listed in Supplementary Table 1. Seven genomes (six *P. luteola* strains and *P. stutzeri* NP_8HO) did not belong to the *P. stutzeri* complex. *P. stutzeri* complex strains had been isolated from different sources, e.g., soil, saline and clinically relevant environments. Overall, the genome size of members of the *P. stutzeri* complex ranged from 3.674 to 5.319 Mb, with an average value of 4.566 Mb, and their GC contents ranged from 59.6 to 65.18%, with an average value of 63.12%. Strains of this species complex have an average of 4,129 CDSs (excluding pseudo-CDSs) (Supplementary Table 1).

Among 123 genomes, we identified 25 novel members of the *P. stutzeri* complex (Supplementary Table 1), accounting for more than 20% of the total number of *P. stutzeri* complex genomes. This result provides evidence of mistakes in the taxonomy and nomenclature of the *P. stutzeri* complex when data were submitted to the NCBI genome database. The novel *P. stutzeri* complex genomes encompassed all sequenced strains of four species [*Pseudomonas kunmingensis* (Xie et al., 2014), *P. saudiphocaensis* (Azhar et al., 2017), *P. songnenensis* (Zhang et al., 2015b) and *P. zhaodongensis* (Zhang et al., 2015a)] and 16 *Pseudomonas* strains without species assignment. The reason for a large proportion of *P. stutzeri* complex genomes being deposited at NCBI with incorrect taxonomy and nomenclature most likely stems from their extreme genetic diversity and a high similarity among their 16S rRNA gene sequences. Our results also suggest that it is hard to determine the taxonomy and nomenclature for certain novel *P. stutzeri* complex strains, which show distant relationships with known *P. stutzeri* complex genomes. The results presented here suggest that FastANI in combination with phylogenetic analysis is a promising approach for correcting and updating the nomenclature to curtail the promulgation of inaccurate information. FastANI is a fast method for calculating ANI for large numbers of genomes (Jain et al., 2018). We set an ANI value of 80% as the threshold for filtering, which may have caused us to miss some *P. stutzeri* complex strains if genomes of novel genomovars are not included in the reference genomes. However, it will not happen as ANI values between genomes of different genomovars were greater than 80% (Supplementary Table 3).

### Genome Similarity and Phylogenetic Relationships

**FIGURE 1:**
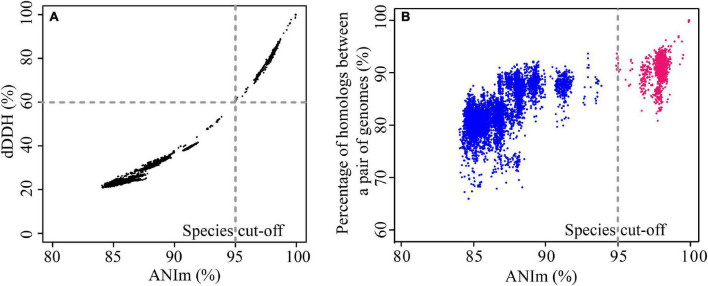
Comparison of similarities and diversities for 123 *P. stutzeri* group genomes. **(A)** Correlation between ANI and dDDH. Each data point represents the dDDH value between two strains against their corresponding ANIm value. The two approaches revealed a significant correlation, with an r^2^ of 0.986. **(B)** Correlation between conserved genes and evolutionary distance for bacterial species. Each data point represents the percentage of homologous genes between two strains plotted against their evolutionary distance, measured as dDDH between the strains. Red points denote pairs of strains that belong to the same genomovar, and blue points denote pairs of strains belonging to different genomovars, according to the species definition standard in this study. Although there is significant correlation, with an r^2^ of 0.738, the pairwise percentage similarities of homologs are discordant with the genome phylogeny.

We evaluated the pairwise percentage similarities of 123 genomes using two different bioinformatics approaches, dDDH and ANI. Use of these two approaches has been proposed to overcome the challenges of conventional laboratory-based DDH for evaluating the overall similarity of bacterial genomes (Colston et al., 2014). dDDH and ANI use different algorithms for the calculations (Colston et al., 2014); however, the results obtained here were highly concordant with each other (Figure 1A), and detailed data are shown in Supplementary Table 3. The r^2^ value was 0.986 for the entire data set, demonstrating that results from the two approaches show good congruence and either method can be used for determining overall genome similarities. Usually, two genomes belonging to the same species would have an ANI cutoff of 94-96% (Konstantinidis and Tiedje, 2005; Richter and Rossello-Mora, 2009), which corresponds to the 70% DDH and 70% dDDH. However, for pairs of a few genomes in this study (mainly belonging to gv 7 and gv 18, see below), we found ANI values of around 95% (with TETRA values > 0.99) corresponded to approximately 60% dDDH, noticeably lower than the expected dDDH values. For instance, *P. stutzeri* DCP-Ps1 and *Pseudomonas* sp. JL972 have an ANI value of 94.89% and dDDH value of 59.9% [95% confidence interval (CI), 57.1–62.7%] (Supplementary Table 3). This result indicated that ANI values were positively correlated with dDDH values, yet an ANI cut-off of around 95% did not correspond to an absolute dDDH value. A previous study reported that the value of 70% DDH could not be used as absolute boundary, but still a gap between 60 and 70% similarity seemed to embrace clear-cut clusters of organisms; given the large extent of diversity among prokaryotes, the circumscription of each genospecies would, in addition, be dependent on each group being studied (Richter and Rossello-Mora, 2009). Correspondingly, NCBI maintains ANI species cutoff values on a species-specific basis, which span much more (or much less than) the default rule of thumb 96% ANI (Federhen et al., 2016). In addition, the phylogenetic tree based on MLSA analysis confirmed that these genomes belonged to the same genomovar since they clustered with the well-characterized strains of *P. stutzeri* complex gv 7 or gv 18 (Supplementary Figure 2). Altogether, for *P. stutzeri* complex, at the ≈ 95% ANI value (Jain et al., 2018) and ≈ 60% dDDH values cutoff, 123 genomes in our study could be assigned into 27 genomovars (referred to as gv). For each genomovar containing at least three genomes, the range of ANI and dDDH is listed in Table 1.

**TABLE 1:** The range of ANI and dDDH values within each genomovar.

Genomovar*	ANIm		dDDH	
	Minimum value (%)	Maximum value (%)	Minimum value (%)	Maximum value (%)
gv 1	97.31	99.96	73.4	100
gv 2	97.67	97.74	77.9	78.9
gv 3	96.51	99.92	69.6	98.8
gv 5	96.54	98.38	68.6	86.1
gv 6	98.45	98.62	84.6	87.6
gv 7	94.89	98.51	59.9	87.6
gv 8	96.91	98.95	72.4	91.5
gv 14	96.65	98.68	70.7	88.6
gv 15	97.25	97.47	75.5	77.5
gv 18	95.57	99.56	62.7	97.7
un 1	98.22	98.35	83.5	85

*Each genomovar containing at least three genomes is involved in statistical analysis.

**FIGURE 2:**
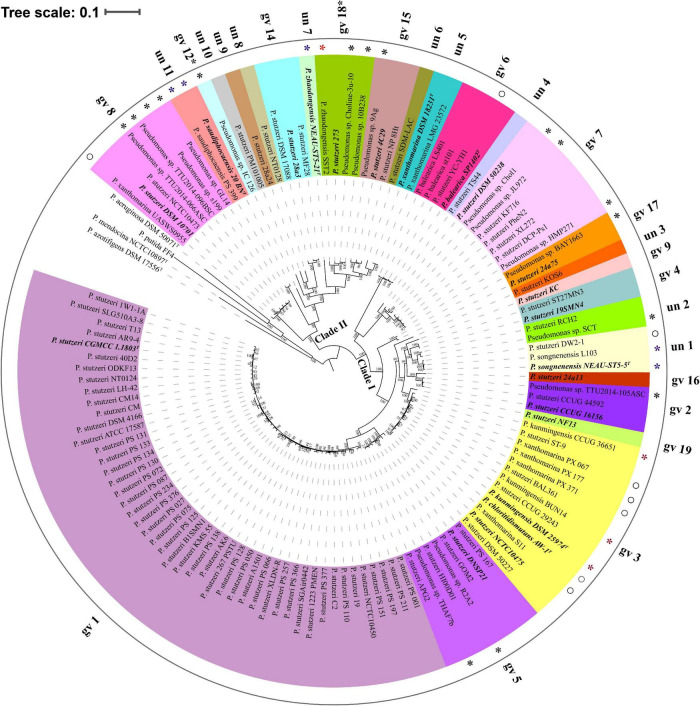
Phylogenetic tree based on the maximum-likelihood method implemented in RAxML. The phylogenetic tree was reconstructed using 524 single-copy orthologous genes shared by 123 *P. stutzeri* strains and four outgroup strains (*P. aeruginosa* DSM 50071^T^, *P. azotifigens* DSM 17556^T^, *P. mendocina* NCTC10897^T^ and *P. putida* FF4). This tree was re-rooted with four outgroup strains using iTOL. 27 groups are marked with different colors and numbers. Type strains and the reference strains of each genomovar are marked with bold italic font. Asterisks denote 25 newly identified members of *P. stutzeri*; blue asterisks represent newly identified genomovars of *P. stutzeri*, and red asterisks and hollow circles denote strains with mistakes in nomenclature. Scale bar corresponds to 0.1 estimated nucleotide substitutions per site.

Based on reference strains of defined genomovars, 82.93% genomes (102 out of 123) belonged to 14 known genomovars. gv 1 was the largest group, comprising 47 genomes, followed by gv 3 (13 genomes), gv 7 (8 genomes), gv 8 (7 genomes), gv 5 (7 genomes), gv 6 (4 genomes), gv 2 (3 genomes), gv 14 (3 genomes), gv 15 (3 genomes), gv4 (2 genomes) and gv 17 (2 genomes); gv 9, gv 16, and gv 19 contained only one strain, respectively (Lalucat et al., 2006). In addition, according to results of MLSA and ANI analysis (Supplementary Figure 2 and Supplementary Table 3), five genomes were most likely assigned to gv 12 and gv 18. For example, four strains (*P. stutzeri* 273, *Pseudomonas* sp. 10B238, *Pseudomonas* sp. Choline-3u-10 and *P. zhaodongensis* SST2) were grouped with representative strain *P. stutzeri* MT-1 of gv 18; they should be members of gv 18. The remaining 11 branches each represented an unknown or novel genomovar (Figure 2). Taken together, these results highlight the extremely high level of genetic heterogeneity within the *P. stutzeri* complex. It speculates that the study of more genomes will uncover more genomovars (Scotta et al., 2013).

To infer the phylogenetic relationship for *P. stutzeri* complex strains, we built both ML (Figure 2) and NJ (Supplementary Figure 3) phylogenetic trees based on concatenated single-copy core genes, using four closely related strains as an outgroup. These two trees showed accordance with each other in strain grouping (*P* < 0.05) by Kendall-Colijn test with λ = 0 implemented in R package Treespace (Jombart et al., 2017). Their topology was also similar with that of the phylogenetic tree reconstructed using the GGDC distance matrix (Kendall-Colijn test, *P* < 0.05) (Supplementary Figure 4). Overall, *P. stutzeri* complex strains could be divided into two major clades (Figure 2). Clade I comprised 96 genomes covering 15 genomovars. Most of the known genomovars were located in this clade, e.g., gv 1 and gv 5. These two genomovars were most closely related to each other. Interestingly, blastp analysis using the *nifH* gene (encoding nitrogenase Fe protein) as a query indicated that all 16 strains possessed the potential ability to fix nitrogen. These strains all belonged to Clade I, mainly belonging to gv1 (Supplementary Table 4). Clade II had 27 genomes covering 12 genomovars; more than half of the unknown genomovars belonged to this clade. In good accordance with the results of ANI and dDDH, the phylogenetic tree based on core genes divided all genomes of the *P. stutzeri* complex into 27 branches (marked with different colors in Figure 2), and members of the same genomovar clustered together on the same branch (Figure 2). Hence, we conclude that the core-gene-based tree reflected the actual affiliations among strains of the *P. stutzeri* complex. Our results also robustly support that 25 newly identified genomes belong to *P. stutzeri* complex, since these strains were mixed well with known *P. stutzeri* complex strains in the tree, with most of them located in known genomovars (Figure 2 and Supplementary Figure 2), e.g., *P. kunmingensis* strains. By contrast, in previous studies, phylogenetic analyses of the 16S rRNA gene and four housekeeping genes (ITS1, *gyrB*, *rpoB* and *rpoD*) distinctly placed *P. stutzeri* complex and *P. balearica* on different phylogenetic branches (Bennasar et al., 1996; García-Valdés et al., 2010). It can be concluded that the phylogenetic tree based on the whole-genome sequences gives much better resolution than a phylogeny based on several housekeeping genes.

### Taxonomic Assignments of *Pseudomonas stutzeri* Complex Strains

In this study, a combination of three different approaches (dDDH, ANIm and core-gene-based phylogenomic tree) allowed us to obtain a high-resolution phylogeny of *P. stutzeri* complex strains. We revealed that 28 strains are inconsistently named among 98 publicly available and 25 newly identified *P. stutzeri* complex genomes analyzed in this study.

Our deep bioinformatics analysis allowed us to accurately assign some strains with uncertain species identification to the species level. In total, there were 16 strains without species names, distributed in nine genomovars, including eight known genomovars. *P. stutzeri* CCUG 16156 is the first sequenced representative of gv 2 and a model organism for denitrification studies (Pena et al., 2012). *Pseudomonas* sp. TTU2014-105ASC and strain CCUG 16156 had an ANIm value of 97.7% and a dDDH value of 77.9%, demonstrating that strain TTU2014-105ASC should belong to gv 2. The core-gene phylogeny placed *Pseudomonas* sp. R2A2 and *Pseudomonas* sp. THAF7b within gv 5 group. ANI values between these two strains and the gv 5 strain *P. stutzeri* DNSP21 were > 96.5%, while the dDDH values were > 68.8%. Taken together, the data strongly support assigning strains R2A2 and THAF7b to gv 5. Three strains (*Pseudomonas* sp. HMP271, JL972 and Chol1) had ANI values of ≥ 95.9% and dDDH values of ≥ 65.8% to strain DSM 50238, the reference strain of gv 7. Four strains, *Pseudomonas* sp. GL14, s199, TTU2014-096BSC and TTU2014-066ASC clustered with *P. stutzeri* DSM 10701, which is the reference strain of gv 8 and considered as a model organism for natural transformation. These strains shared ANIm and dDDH values with DSM 10701 of ≥ 97.6% and ≥ 78.7%, respectively. *Pseudomonas* sp. 9Ag belongs to gv 15, since it had an ANI value of 97.3% and a dDDH value of 75.9% to *P. stutzeri* 4C29, the reference strain of gv 15. *Pseudomonas* sp. BAY1663 was assigned to gv 17, as it had an ANIm value of 98.04% and a dDDH value of 82.2% with the gv 17 strain *P. stutzeri* 24a75 (Lalucat et al., 2006). According to MSLA, ANI and dDDH analyses, *Pseudomonas* sp. 10B238 and Choline-3u-10 belonged to gv 18, and *Pseudomonas* sp. IC_126 affiliated to gv 12. *Pseudomonas* sp. SCT and *P. stutzeri* RCH2 grouped together in unknown genomovar un 2.

In addition, our results revealed mistakes in the nomenclature of 12 strains, especially seven *P. xanthomarina* strains, which were assigned into three different genomovars. Group un 5 represented the actual *P. xanthomarina* species, as the type strain *P. xanthomarina* DSM 18231^T^ was located on this branch. *P. xanthomarina* UASWS0955 and *P. stutzeri* DSM 10701 had an ANIm value of 97.2% and a dDDH value of 74.7%. Therefore, *P. xanthomarina* UASWS0955 should be re-assigned as *P. stutzeri* UASWS0955. Four *P. xanthomarina* strains, S11, PX_371, PX_177 and PX_067, belong to gv 3, as they had minimum ANIm of 96.8% and dDDH of 71.1% with *P. stutzeri* CCUG 29243, the reference strain of gv 3. Surprisingly, a number of taxonomic controversies existed within the gv 3 group. This group contained two newly described species, *P. kunmingensis* [type strain *P. kunmingensis* DSM 25974^T^ (Xie et al., 2014)] and *P. chloritidismutans* [type strain *P. chloritidismutans* AW-1^T^ (Wolterink et al., 2002)]; however, the ANI and dDDH for any pair of genomes in this group exceeded the specified threshold for species delineation. This result was in accordance with previous reports for *P. chloritidismutans* (Wolterink et al., 2002; Cladera et al., 2006; Scotta et al., 2013; Mehboob et al., 2016). *Pseudomonas chloritidismutans* has been identified as a novel species because strain AW-1 can dissimilate perchlorate or chlorate, which was not found in *P. stutzeri* DSM 50227, the reference strain of gv 3. However, this unique feature is most likely a result of lateral gene transfer in the environment; additionally, dissimilation of perchlorate or chlorate is also found in strains belonging to gv 1, gv 3 and gv 5 (Cladera et al., 2006). One crucial reason for describing *P. kunmingensis* as a novel species was that *P. kunmingensis* DSM 25974^T^ has a DDH value of 38.92% (± 3.44%, standard deviation of three replicates) with *P. stutzeri* ATCC 17588^T^ (= CGMCC 1.1803^T^), the reference strain of gv 1 (Xie et al., 2014). In fact, both *P. kunmingensis* DSM 25974^T^ and BUM14 have dDDH values of > 70% and ANI values of > 95% with the strains of the gv 3 branch. Therefore, we suggest that a mistake occurred in the nomenclature for *P. chloritidismutans* and *P. kunmingensis*, and representatives of these two taxa should belong to the same species. Each pair of genomes among *P. stutzeri* DW2-1, *P. songnenensis* L103 and *P. songnenensis* NEAU-ST5-5^T^ had an ANIm value > 98% and dDDH > 83%. Hence, *P. stutzeri* DW2-1 should be renamed *P. songnenensis* DW2-1. In the gv 6 group, *P. stutzeri* YC-YH1 and three *P. balearica* strains (LS401, st101 and DSM_6083^T^) grouped together, supporting assignment of *P. stutzeri* YC-YH1 to *P. balearica*. Our results showed that *P. zhaodongensis* SST2 and *P. zhaodongensis* NEAU-ST5-21^T^ belong to different genomovar branches, as the shared ANI and dDDH values between them greatly below the species cutoff value. This demonstrated mistaken taxonomic nomenclature for *P. zhaodongensis* SST2. Our results could assign some groups to valid species. For example, un 11 contained two strains, the type strain *P. saudiphocaensis* 20_BN^T^ and *P. saudiphocaensis* PS_399, suggesting that un 11 should represent *P. saudiphocaensis*.

Collectively, our results conclude that either of the ANI and dDDH indexes enables robust determination of genomic taxonomy for prokaryotic species. However, ANI is much appropriate for re-evaluating the taxonomy of *P. stutzeri* complex, since this species complex has ANI genomovar cut-off for set at approximately 95%, but dDDH genomovar cutoff value greatly below 70% (Meier-Kolthoff et al., 2013; Colston et al., 2014). Furthermore, dDDH can only be calculated through a Web-based interface that requires manually uploading genome sequences, while ANI can be easily analyzed through many standalone tools, such as Jspecies (Richter and Rossello-Mora, 2009), OrthoANI (Lee et al., 2016) and OrthoANIu (Yoon et al., 2017). In addition, NCBI developed a protocol for using ANI genome neighboring statistics in conjunction with reference genomes from type and proxytype to improve taxonomic assignments (Federhen et al., 2016; Ciufo et al., 2018). Consequently, we recommend ANI as it is more time-effective when it comes to large comparisons (Richter and Rossello-Mora, 2009; Colston et al., 2014).

### Pan- and Core-Genome Within *Pseudomonas stutzeri* Complex Genomes

**FIGURE 3:**
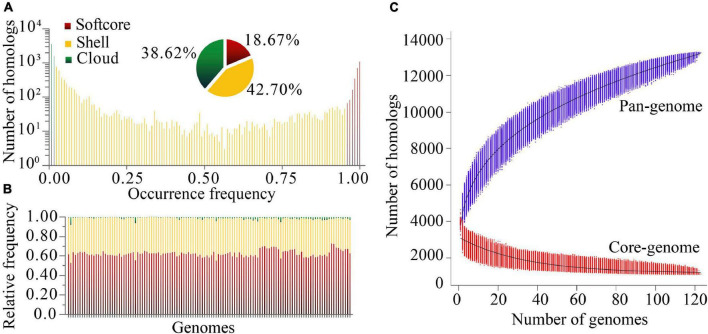
Homologous gene cluster based pan-genome. **(A)** Composition of the *P. stutzeri* pan-genome. **(B)** Relative frequency of softcore, shell and cloud pan-genes for individual genomes; on the X-axis, each vertical bar represented one genome. **(C)** Simulations of the increase of pan-genome size and decrease of core-genome size. Genomes were sampled as 20,000 random combinations of each given number of genomes. Solid black lines indicate pan- and core-genome curves fitted using points from all random combinations according to the models implemented in PGAP software.

Based on protein sequence similarity analysis using OrthoFinder, the 123 *P. stutzeri* complex genomes possessed a pan-genome size of 13,261 homologous protein clusters. We categorized clusters in the pan-genome according to their frequency of presence (Koonin and Wolf, 2008): 1,104 strict core gene clusters shared by all genomes, 2,476 softcore gene clusters shared by more than 95% genomes (118–123 genomes), 5,663 shell gene clusters shared by 2–95% genomes (3–117 genomes), and 5,122 cloud gene clusters present in only one or two genomes (Figure 3A). This classification system can overcome some of the technical uncertainty, particularly for the softcore, where strict core genes could be placed if they were missed from draft genomes (Gordon et al., 2017). The strict core genes and softcore genes constituted only 8.33% and 18.67% of the pan-genome, respectively. These analyses may lead to better understanding of the minimal set of genes for *P. stutzeri* complex. The relatively small size of the core genome reflects the high genetic diversity of *P. stutzeri* complex. Of the pan-genome clusters, 81.3% belonged to accessory genes (shell and cloud), which may be vital for this species in occupying diverse habitats and ecological niches. The high fraction of shell genome (42.7% overall) is key for understanding genome dynamics of *P. stutzeri* complex because it reflects how genes present at intermediate frequencies drive adaptation of species. Furthermore, a previous study showed that proportion of shell genes is independent of genome size (Gautreau et al., 2020). Relative proportions of the three gene categories per genome were relatively constant within *P. stutzeri* complex. On average, each individual genome was composed of 62.80% ± 3.32% (mean and standard deviation) softcore pan-genes, 35.83 ± 3.01% shell genes and 1.37 ± 1.20% cloud genes (Figure 3B). Several genomes had more than twice (and even four times, e.g., *P. stutzeri* strain B1SMN1 and *P. stutzeri* 19) the average proportion of cloud genes. It appeared to be partly explained by a large genome size for these genomes, driven by large gene gain and small gene loss events. For example, of 123 genomes, *P. stutzeri* strain B1SMN1 has the largest genome size, encompassing many more genes, and recently experienced 859 gene gain and 77 gene loss events (see below).

Modeling of pan-genome size by iterative random sampling of genomes showed that members of *P. stutzeri* displayed an open pan-genome. The gene accumulation curve for an increasing number of genomes fitted the power law, *y* = *ax*^0.316^ + *b* (95% confidence interval for a = 2630.59 ± 1.21, and b = 1166.33 ± 5.14, R-square of curves fitting = 0.9998) (Figure 3C). Approximately 31 to 644 new genes would be added to each new genome, resulting in an open pan-genome whose size shows a tendency to increase with the addition of new genomes. An open pan-genome is likely to be typical in species that colonize multiple environments and have multiple ways of exchanging genetic material. It is largely responsible for the ability of *P. stutzeri* complex to adapt to different ecological niches. The gene occurrence plot showed a closed core-genome with a finite number of core genes for *P. stutzeri* complex, which fitted well with the exponential law, *y* = *ae*^−0.03*x*^ + *b* (95% confidence interval for a = 1980.84 ± 7.00, and b = 1147.46 ± 2.31, R-square of curves fitting = 0.9582) (Figure 3C).

We characterized the functions of pan-genome genes using COG functional categories (Galperin et al., 2019). The core pan-proteomes (strict core and softcore) carried a large proportion of genes involved in J (Translation, ribosomal structure and biogenesis), E (Amino acid transport and metabolism), and M (Cell wall/membrane/envelope biogenesis) (Fisher’s exact test, *p* < 0.001, Supplementary Figure 5). The accessory pan-proteomes (shell and cloud) had more than twice the number of genes associated with categories X (Mobilome: prophages, transposons) and V (Defense mechanisms) compared with their respective core pan-proteomes (Fisher’s exact test, *p* < 0.001). These two functional categories reflect the dynamics of *P. stutzeri* complex genomes (Richter and Rossello-Mora, 2009; Galperin et al., 2019). Functional analysis of the pan-genome showed that approximately 45% of pan-genome genes (5907/13261) do not match any sequence present in the COG database for *P. stutzeri* complex. These poorly characterized genes mainly belonged to the cloud genes (3719 out of 5122) and shell genes (1974 out of 5663). The proportion of poorly characterized genes is larger than that in *P. aeruginosa* (Freschi et al., 2019). These results highlighted that our knowledge of the functions of *P. stutzeri* complex genes is still far from being complete, and more investigations need to be performed for this species complex.

### Size Variation of Gene Families

**FIGURE 4:**
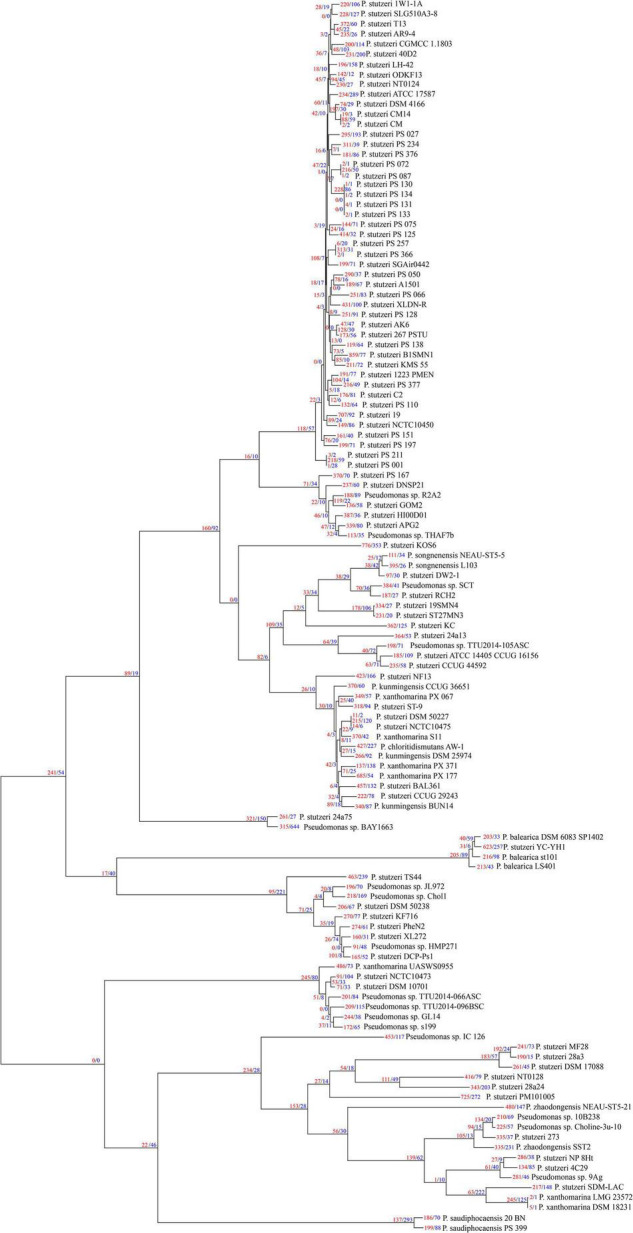
*P. stutzeri* genome dynamics. Estimated events of genome content evolution were mapped onto the ML phylogenic tree, which was generated by removing the four outgroup strains (*P. aeruginosa* DSM 50071^T^, *P. azotifigens* DSM 17556^T^, *P. mendocina* NCTC10897^T^ and *P. putida* FF4) of Figure 2 using iTOL. Numbers on branches indicate numbers of genes gained (red) and lost (blue). The tree scale is in the units of the number of nucleotide substitutions per site.

We used Count software with a birth-death likelihood model to delineate the dynamics of genome content across the genealogy of *P. stutzeri* complex. Results revealed substantial gene gain and loss events occurred in *P. stutzeri* complex genomes (Figure 4). Most of the gene gain events occurred in genes belonging to accessory clusters (shell and cloud genes). In contrast, nearly all gene loss events occurred in genes belonging to shell and softcore categories (Supplementary Table 5). This demonstrates that shell genes are the result of complex histories characterized by frequent events of gain and losses (Brito et al., 2018). Compared with internal phylogenetic nodes, most gene gain and loss events occurred at terminal nodes. This result is fully compatible with previous findings indicating that genes are gained and lost at higher rates on the tips of phylogenetic trees (Hao and Golding, 2006). Overall, gain of gene families dominates the evolution of *P. stutzeri* complex, occurring at more than three times the rate of loss. This suggested that gene gains contribute more in driving the divergence of gene content and pan-genome expansion. High rates of gene gain appear to coincide with the ability for natural transformation in many *P. stutzeri* complex strains. Nine strains had suffered from gene gain/loss events affecting more than 16% gene content: *P. stutzeri* B1SMN1, *P. stutzeri* 19, *P. stutzeri* KOS6, *P. chloritidismutans* AW-1, *P. xanthomarina* PX_177, *Pseudomonas* sp. BAY1663, *P. stutzeri* YC-YH1, *P. stutzeri* TS44 and *P. stutzeri* PM101005. Bacterial genomes can be highly variable, with loss and gain of genes greatly affecting the evolution of bacterial populations. A previous study using mathematical modeling showed that extensive gene gain and loss within the accessory genome pushes prokaryotes beyond the homologous recombination barrier and accelerates genome sequence divergence, resulting in speciation (Iranzo et al., 2019). We conclude that gene gain and loss is the central factor driving the genetic diversity of *P. stutzeri* complex and the subsequent adaptation of this species complex to diverse ecological niches (Rodriguez-Valera et al., 2016).

When tracing the origin of cloud genes, Blastp analysis querying the NCBI non-redundant database identified best hits for 4225 out of 5122 genes (Table 2). The taxonomy of the best hits indicated that 96.47% of cloud genes had a close homolog within the phylum Proteobacteria, 73.25% within the genus *Pseudomonas*. These results showed that most genes acquired were obtained from closely related species through horizontal gene transfer (HGT). We also identified 88 genes obtained from distant taxa. These genes have closest homologs in other phyla, mainly Firmicutes (18 genes) and Bacteroidetes/Chlorobi group (10 genes) (Table 2). These genes were most likely obtained through HGT, allowing their hosts to exploit new habitats (Moulana et al., 2020). In addition, we found no significant blastp hits for 897 genes in the NCBI non-redundant database, representing 17.51% of the cloud genome. This result is compatible with the observation that a large proportion of cloud genes could not be assigned to any known COG functional category. No significant blastp hits for many cloud genes could be explained that as with the large amount of genomic data available today, many genes are uncharacterized.

**TABLE 2:** Taxonomic characterization of best blastp hits for cloud genes.

Taxonomic status					Number	
Archaea						1
Eukaryota						7
Viruses						11
Unclassification						42
Bacteria	other phyla					88
	Proteobacteria					4,076
		γ-proteobacteria				3,666
			Pseudomonadales			3,215
				Pseudomonadaceae		3,166
					Pseudomonas	3,095

### Genotypic Diversity Within *Pseudomonas stutzeri* Complex Genomes

We used the fraction of homologs shared between pairs of genomes to reflect genetic diversity. As shown in Figure 1B. We found that the fraction of homologs correlates (Spearman’s ρ = 0.77, *P* = 2.2e-16) with the evolutionary distance measured by ANI (Konstantinidis and Tiedje, 2005), which is consistent with results reported previously based on other bacterial groups (Konstantinidis et al., 2006, 2009; Jain et al., 2018). To a large extent, the genotypic similarity between two genomes may be predicted from their evolutionary distance. However, a larger correlation between percentage of homologs and ANI was observed for pairs of genomes from different genomovars (Spearman’s ρ = 0.63, *P* = 2.2e-16) than those within the same genomovar (Spearman’s ρ = 0.22, *P* = 1.858e-15). Meanwhile, overall, pairs of genomes within the same genomovar had significantly higher fractions of homologs than those within different genomovars (*P* < *0.01*). Correspondingly, strains within the same and different genomovars differed in 0.04–19.22% and 6.42–34.17% of their total genes, respectively, revealing extensive genetic diversity within *P. stutzeri* complex. The fraction of homologs between and within genomovars partially overlapped. For inter-genomovar genomes, some pairs of genomes with less than 95% identity by ANI showed relatively large percentages of homologs, e.g., > 90%. This may be due to close affiliations among these strains, as most of these genomes clustered together in Clade II and had short branch lengths compared with other genomes. Some pairs of genomes within the same genomovar had a relatively low percentage of homologs, mainly due to the high frequency of gene gain and loss events occurring in these genomes, such as *P. stutzeri* B1SMN1 and *P. stutzeri* 19 in gv1, and *P. chloritidismutans* AW-1 and *P. xanthomarina* PX_177 in gv3. For instance, *P. stutzeri* B1SMN1 obtained 859 genes and lost 77 genes, accounting for 17.25% and 1.55% of its total predicted protein-coding genes, respectively (Figure 4). Extensive gene gain and loss events occurring in *P. stutzeri* complex genomes produced inconsistencies in the relationship between the fraction of homologs and evolutionary relatedness.

We speculate that the high genetic diversity reflects the many and different ecological niches potentially occupied by members of *P. stutzeri* complex, and that the genomic groups that have been described so far are evolving in a speciation process that may give rise to 27 novel species from a genomic point of view.

### Distribution of the Ectoine Biosynthesis Gene Cluster in *Pseudomonas stutzeri* Complex

**FIGURE 5:**
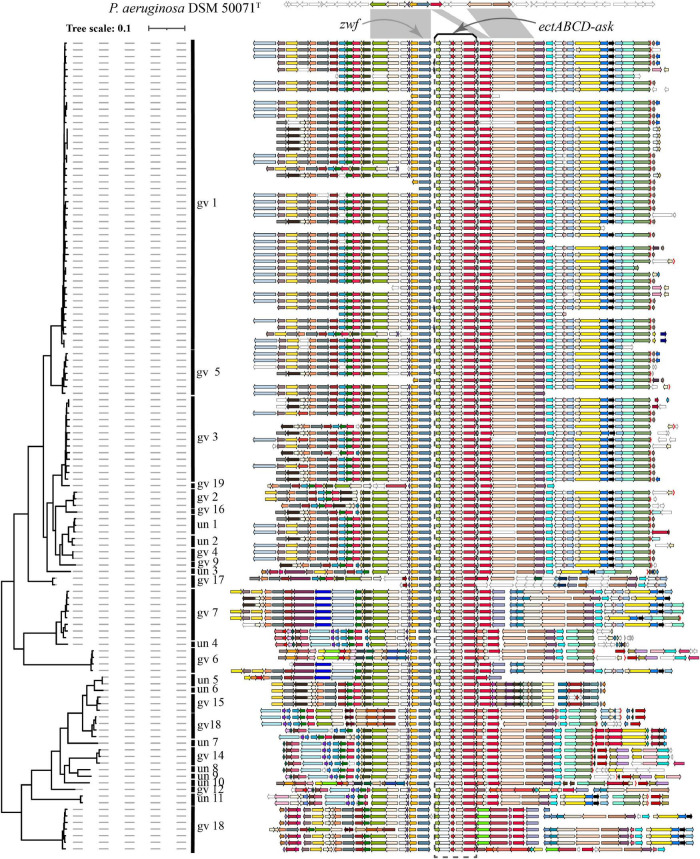
Visualization and mapping of the ectoine biosynthesis gene cluster (*ectABCD-ask*) and its flanking genomic elements (20 genes) to Figure 2 in 123 *P. stutzeri* complex genomes. Gcluster was used to visualize and map the *ect* cluster to the phylogeny shown in Figure 2, displayed in a rectangular shape. Gcluster was run using three inputs, including (1) a directory containing the 123 genomes in GenBank format, (2) a list of genes of interest, in which each row contains a locus tag of the ectC gene from each of the 123 genomes, and (3) a tree file of Figure 2 in Newick format. Then, the generated SVG map was edited by Adobe Illustrator to delete strains name, remove outgroup branches and add the genomovar information. At the top, these contexts are compared with *P. aeruginosa* DSM 50071^T^, which has no *ect* cluster but similar organization of flanking genes in its genomes. Genomic contexts have been reorientated around *ectC*, and parts of flanking genes were found to be missing from certain draft genomes. *ectABCD-ask* is marked by a black rectangular dashed box. Homologous gene clusters are filled in different colors; unique genes, pseudo genes and RNA genes are in white, with black, deep-gray and red borders, respectively.

Blastp analysis showed that out of 9,548 *Pseudomonas* genomes, 553 genomes carried *ectC* genes. However, most of these genomes contained an orphan *ectC*, and only 165 genomes possessed the entire ectoine biosynthesis gene cluster, accounting for 1.73% of the total *Pseudomonas* genomes used in this study (data not shown). It was noted that *ectA* or *ask_ect* gene was missing from some genomes out of 165 genomes. A surprising finding was that all 123 *P. stutzeri* genomes contained the ectoine biosynthesis gene cluster, except for six genomes lacking the *ectA* gene, possibly resulting from microevolution as these genomes were located in the same branch (Figure 5). This result indicated *ect* cluster was mainly distributed in *P. stutzeri* complex, compared with other *Pseudomonas* species. According with above result, many *P. stutzeri* complex strains can grow in high concentrations of NaCl, such as 7.02% (w/v) NaCl for *P. stutzeri* TS44 (Li et al., 2012), 8% (w/v) NaCl for *P. xanthomarina* (Romanenko et al., 2005), and 8.5% (w/v) NaCl for *P. balearica* (Bennasar et al., 1996). The *ect* genes and their flanking genes displayed high levels of similarity and conserved synteny among *P. stutzeri* complex strains. In addition, we observed good coherence between the phylogeny and organization of *ect* genes and corresponding flanking genes (Figure 5). Compared with *P. aeruginosa* DSM 50071^T^, *ect* cluster seems to be obtained by insertion at the site near to *zwf* gene, encoding glucose-6-phosphate dehydrogenase (Ma et al., 1998). We suspect that obtaining the *ect* cluster was an early evolutionary adaptation for the ancestor of *P. stutzeri* complex, after it split from other members of the *Pseudomonas* genus. Taken together, our results provided strong evidences that the ancestor of *P. stutzeri* complex originated from a high-salt habitat.

## Conclusion

Deep bioinformatics is a powerful tool for clarifying the taxonomic status and nomenclature of *P. stutzeri* complex. We revealed that a large proportion of *P. stutzeri* complex genomes (33 out of 123 genomes) are currently misidentified, including 25 genomes submitted as other *Pseudomonas* in the NCBI genome database that actually belong to *P. stutzeri* complex strains, and that 8 out of 98 genomes have the incorrect species name. All 123 sequenced genomes could be ascribed to 27 genomovars, and their affiliations were clearly delineated using a whole-genome-based phylogenetic tree. We observed an open pan-genome and a large proportion of accessory genes for individual genomes of this species. Taken together, our results verify that *P. stutzeri* complex manifested extreme genetic diversity at the genome scale. In accordance with the ability for natural transformation of a substantial number of *P. stutzeri* complex strains, many gene gain and loss events have occurred at the terminal nodes, with a high ratio of gene gain relative to gene loss; this was likely a crucial factor leading to genetic diversity of *P. stutzeri* complex genomes. Gain and loss events greatly influence the coherence between the percentage of homologs between a pair of genomes and their evolutionary distance. We also revealed that all *P. stutzeri* complex genomes harbor an ectoine gene cluster, possibly supporting a high-salt origin for this species.

## Data Availability Statement

The datasets presented in this study can be found in online repositories. The names of the repository/repositories and accession number(s) can be found in the article/Supplementary Material.

## Author Contributions

XL conceived and designed the study and performed the bioinformatic analyses. XL, ZY, WL, and GZ analyzed all the data. XL, ZW, and HY wrote the original draft of the manuscript. XL and ZY supervised the bioinformatic analysis work and reviewed and edited the manuscript. All authors approved the final manuscript.

## Conflict of Interest

The authors declare that the research was conducted in the absence of any commercial or financial relationships that could be construed as a potential conflict of interest.

## Publisher’s Note

All claims expressed in this article are solely those of the authors and do not necessarily represent those of their affiliated organizations, or those of the publisher, the editors and the reviewers. Any product that may be evaluated in this article, or claim that may be made by its manufacturer, is not guaranteed or endorsed by the publisher.

## Footnotes

1
https://www.ncbi.nlm.nih.gov/genome/browse/

2
https://www.ibm.com/aspera/connect/

3
http://ggdc.dsmz.de/ggdc.php

4
https://github.com/Xiangyang1984/P._stutzeri_Complex_Manuscript/blob/main/extract_protein_dir.pl

5
https://github.com/Xiangyang1984/P._stutzeri_Complex_Manuscript/blob/main/format_modification.pl

6
https://github.com/Xiangyang1984/P._stutzeri_Complex_Manuscript/blob/main/Concat_Seq.pl

7
https://github.com/Xiangyang1984/P._stutzeri_Complex_Manuscript/blob/main/COG_annotation.pl
