# Supplementary material for: Comparative Genomics of Pseudomonas stutzeri Complex: Taxonomic Assignments and Genetic Diversity
Source: Front Microbiol. 2022 Jan 13;12:755874. doi: 10.3389/fmicb.2021.755874 (PMC8792951; doi:10.3389/fmicb.2021.755874)
Supplement: Supplementary file 1 [file Data_Sheet_1.pdf]

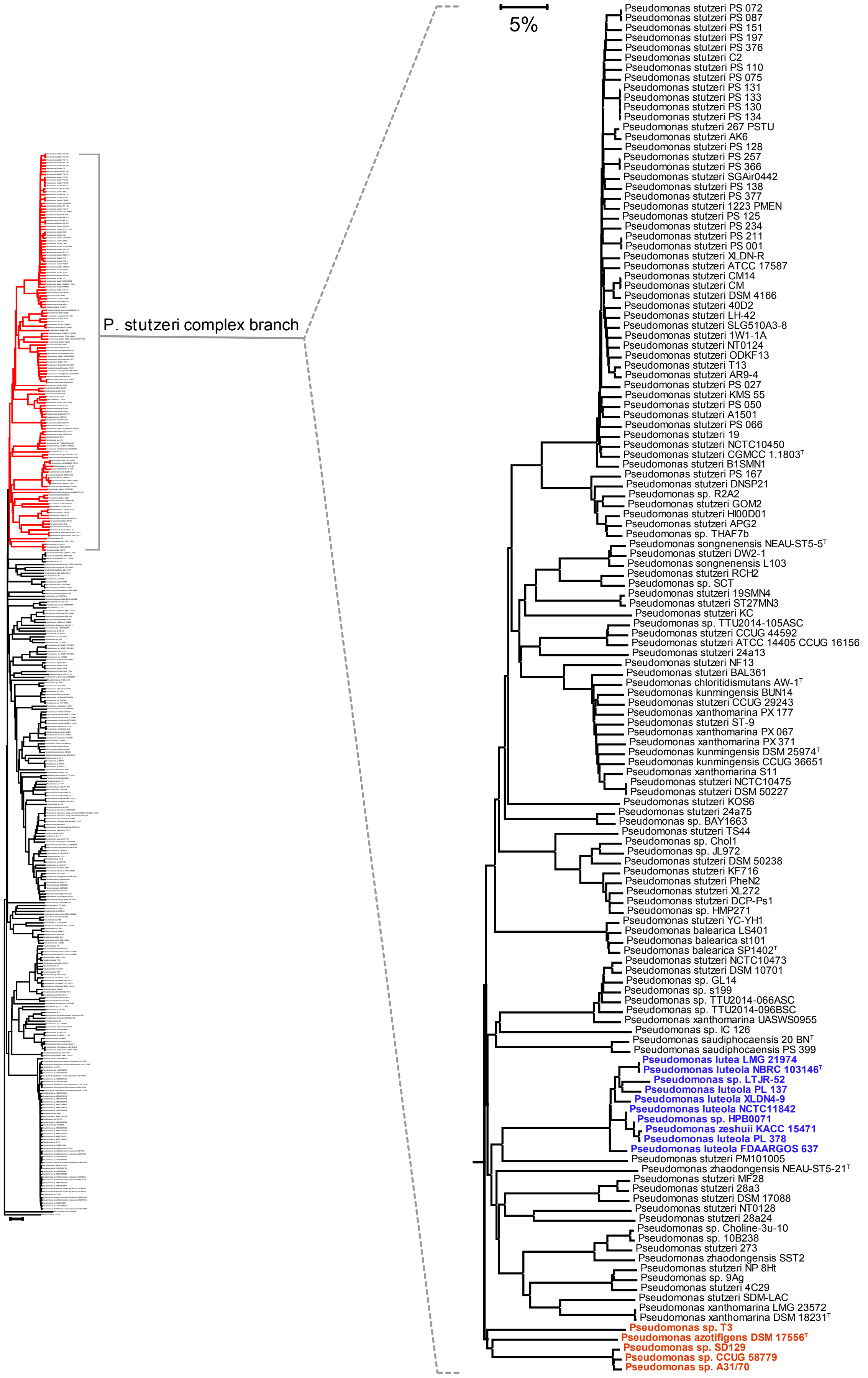

Figure S1. Phylogenetic tree was inferred based on a "100%-ANI" distance matrix using MEGAX with the Neighbor-Joining method. Phylogeny for 368 *Pseudomonas* genomes is shown at the right. The *P. stutzeri* complex branch is shown magnified at the left. Ten strains (six *P. luteola* strains, *P. lutea* LMG 21974, *P. zeshuii* KACC 15471, and *Pseudomonas* sp. strains HPB0071 and LTJR-52) clusted together and were marked with blue bold font. The tree is drawn to scale, with branch lengths in the same units as those of the evolutionary distances used to infer the phylogenetic tree.
