# Supplementary material for: Comparative Genomics of Pseudomonas stutzeri Complex: Taxonomic Assignments and Genetic Diversity
Source: Front Microbiol. 2022 Jan 13;12:755874. doi: 10.3389/fmicb.2021.755874 (PMC8792951; doi:10.3389/fmicb.2021.755874)
Supplement: Supplementary file 2 [file Data_Sheet_2.pdf]

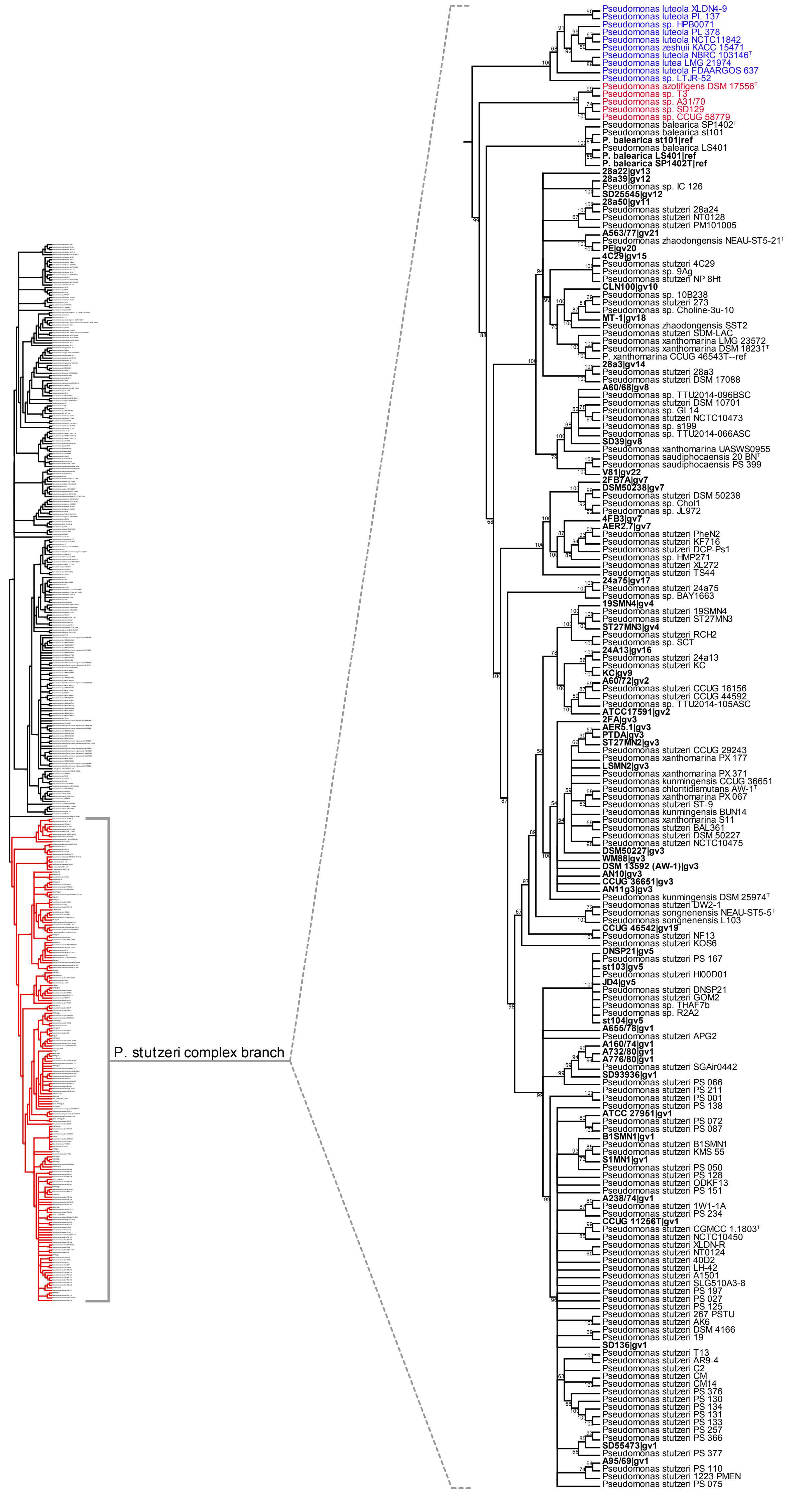

Figure S2. A conservative phylogenetic tree was inferred based on the concatenated analysis of the three genes (16S rRNA, gyrB and rpoD partial gene sequences) using MEGAX with the Neighbor-Joining method. Phylogeny for 420 strains, comprising 361 *Pseudomonas* genomes analyzed in this study, and 53 well-characterized *P. stutzeri* strains plus six outgroup strains (marked with bold font), is shown at the right. The *P. stutzeri* complex branch is shown magnified at the left. Ten strains (six *P. luteola* strains, *P. lutea* LMG 21974, *P. zeshuii* KACC 15471, and *Pseudomonas* sp. strains HPB0071 and LTJR-52) clusted together and were marked with blue bold font. Seven draft *Pseudomonas* genomes, in which 16S rRNA gene or *rpoD* were not identified, were excluded for analysis. All branches have bootstrap value ≥ 50% (based on 1,000 repetitions).
