# Supplementary figures and images for: Comparative Genomics of Pseudomonas stutzeri Complex: Taxonomic Assignments and Genetic Diversity
Source: Front Microbiol. 2022 Jan 13;12:755874. doi: 10.3389/fmicb.2021.755874 (PMC8792951; doi:10.3389/fmicb.2021.755874)

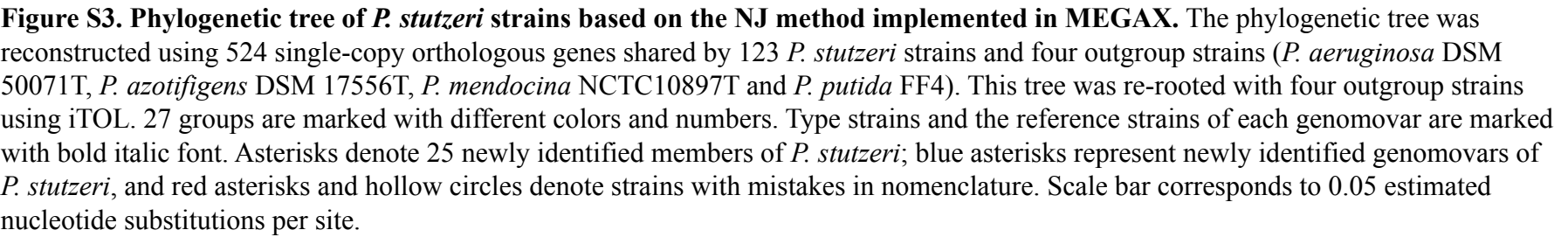

Supplement: Supplementary file 3 [file Data_Sheet_3.pdf]
