# Supplementary material for: Comparative Genomics of Pseudomonas stutzeri Complex: Taxonomic Assignments and Genetic Diversity
Source: Front Microbiol. 2022 Jan 13;12:755874. doi: 10.3389/fmicb.2021.755874 (PMC8792951; doi:10.3389/fmicb.2021.755874)
Supplement: Supplementary file 4 [file Data_Sheet_4.pdf]

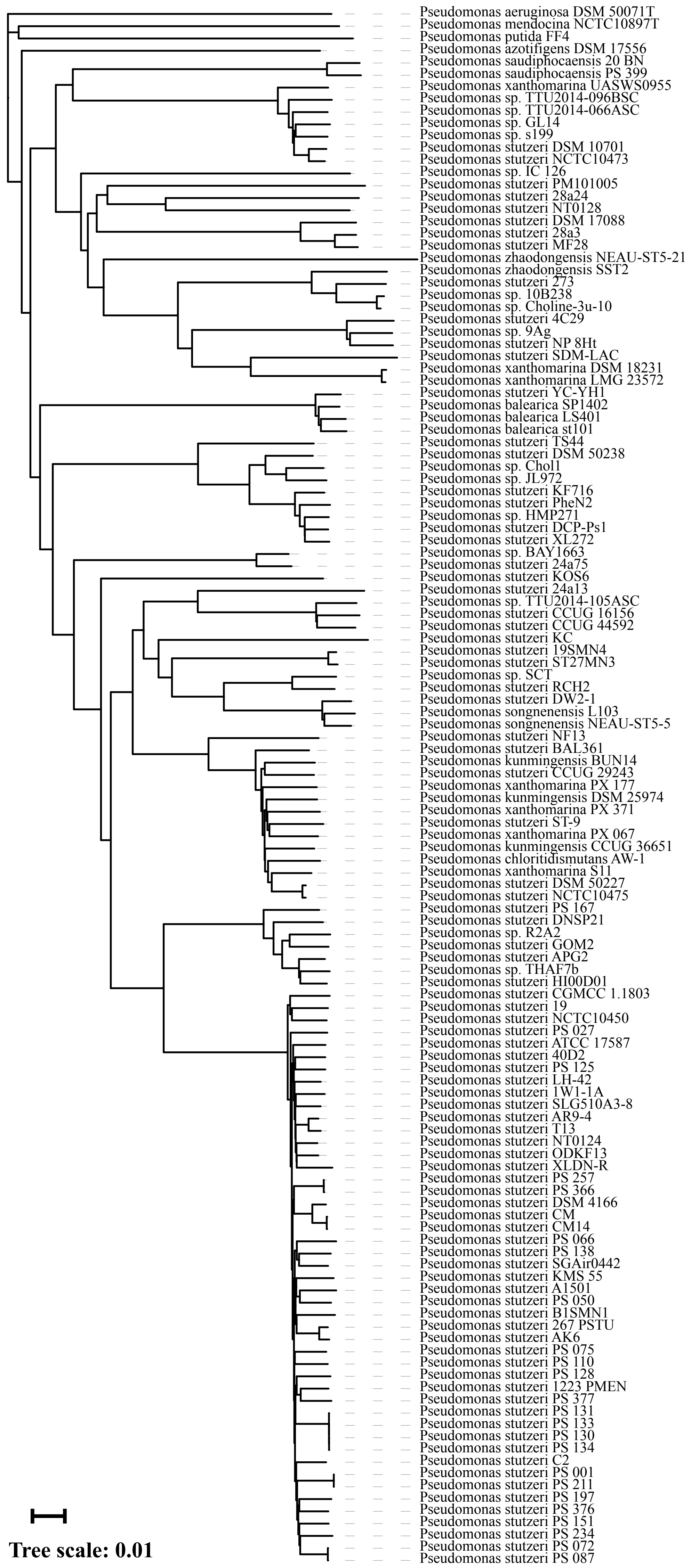

Figure S4. Phylogenetic analysis of *P. stutzeri* genomes using FastME based a matrix of GGDC distance. Four close related strains were used as outgroup. Branch lengths are scaled in terms of GGDC distance. The tree is drawn to scale, with branch lengths in the same units as those of the evolutionary distances used to infer the phylogenetic tree.
