# Supplementary material for: Comparative Genomics of Pseudomonas stutzeri Complex: Taxonomic Assignments and Genetic Diversity
Source: Front Microbiol. 2022 Jan 13;12:755874. doi: 10.3389/fmicb.2021.755874 (PMC8792951; doi:10.3389/fmicb.2021.755874)
Supplement: Supplementary file 5 [file Data_Sheet_5.pdf]

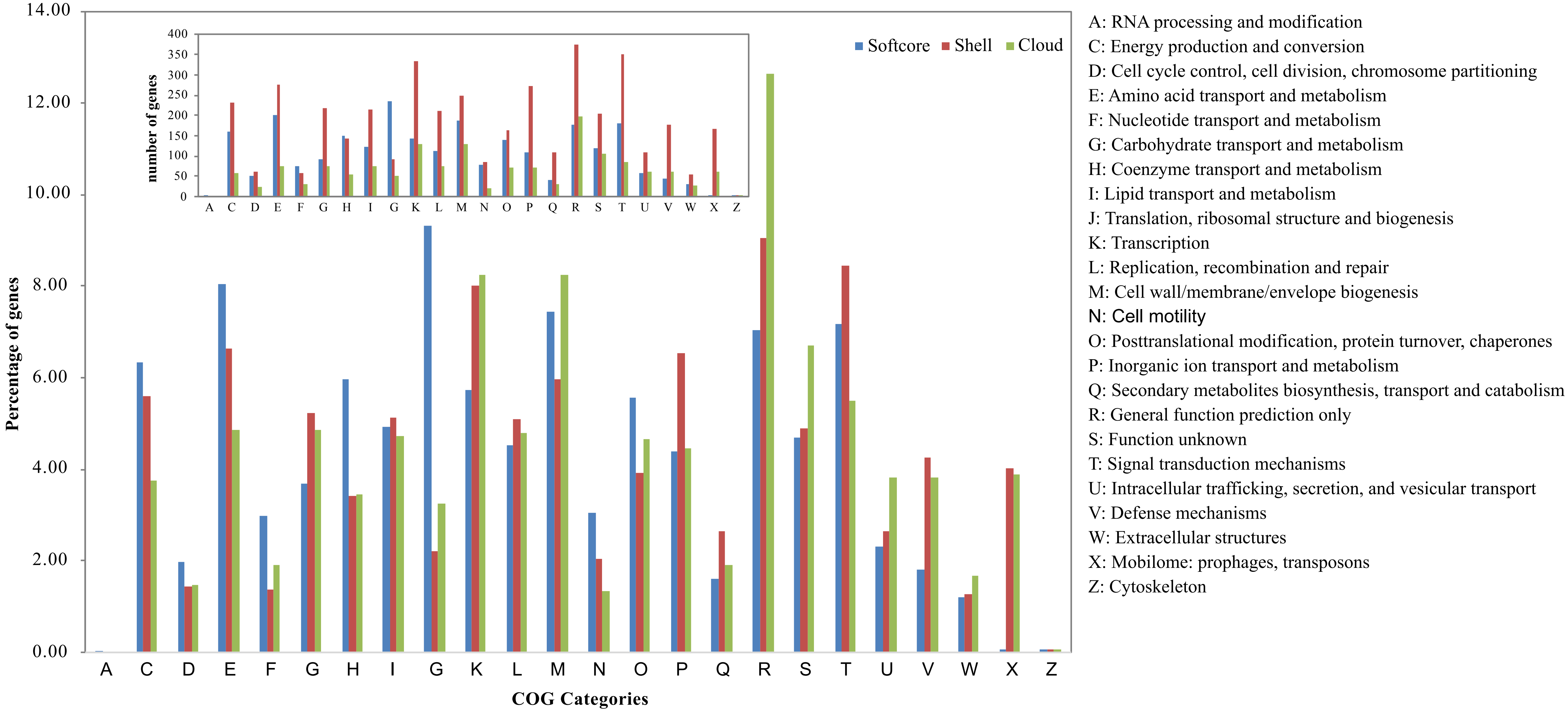

Figure S5. Functional distribution of softcore genes, shell genes and cloud genes in *P. stutzeri* genomes.
